# Supplementary material for: The Role of Stress Hyperglycemia on Delirium Onset
Source: J Clin Med. 2025 Jan 10;14(2):407. doi: 10.3390/jcm14020407 (PMC11766312; doi:10.3390/jcm14020407)
Supplement: Supplementary file 1 [file jcm-14-00407-s001.zip › jcm-3337910-supplementary.pdf]

Study	Case definition adequate	Representativeness of the cases	Selection of controls	Definition of controls	Comparability based on design or analysis	Ascertainment of exposure	Same method of ascertainment for case and controls	Non response rate	Total Score
Bucerius jet al 2003	★	★	★	★	-	-	★	-	5
Bucerius e al 2004	★	★	★	★	-		★	-	5
Bucerius et al 2005	★	★	★	★	-		★	-	5
Yildizeli et al 2017	★	★	★	★	-	-	★	-	5
Kotfis et al 2018	★	★	★	★	★		★		7
Milisen et al 2020	★	★	★	★	★	-	-	-	5
Ordonez-Velasco et al 2021	★	★	★	★	★	-	-	-	5
Li et al 2021	★	★	★	★	★	-	-	-	5
Tan et al 2008	★	★	★	★	★		-	-	5
Nikolic et al 2012	★	★	★	★	★	-	★	-	6
Smulter et al 2013	★	★	★	★	★	-	★	-	6
Ding et al 2024	★	★	★	★	★★	★	★	-	8
Afonso et al et al 2010	★	★	★	★	★	-	★	-	6
Mauri et al 2011	★	★	★	★	★	-	★	-	6
Miyazaki et al 2011	★	★	★	★	★	-	★	-	6
Jodati et al 2013	★	★	★	★	★		★	-	6
Kotfis et al 2019	★	★	★	★	★		★		6
He et al 2021	★	★	★	★	★★	★	★		8
Huang et al 2019	★	★	★	★	★		★	-	6
Yang et al 2020	★	★	★	★	★★	★	★	-	8
He et al 2020	★	★	★	★	★	★	★	-	7
Ma et al 2023	★	★	★	★	★	★	★	-	7
Yang et al 2024	★	★	★	★	★★	★	★	-	8

Wang et al 2018	★	★	★	★	★	-	★	-	6
Ahn et al 2022	★	★	★	★	★		★	-	6
Haynes et al 2021	★	★	★	★	★		★	-	6
Wang et al 2021	★	★	★	★	★		★	-	6
et al 2008	★	★	★	★	★		★	-	6
Venkatarkrishnaiah et al 2022	★	★	★	★	★		★	-	6
Shang et al 2024	★	★	★	★	★★		★	★	8
Shen et al 2022	★	★	★	★	★		★	-	6
Shih et al 2007	★	★	★	★	-		★	-	5
Kolk et al 2022	★	★	★	★	★		★	-	6
Gold et al 2022	★	★	★	★	★		★	-	6
Zheng et al 2024	★	★	★	★	★		★	★	5
Visser et al 2015	★	★	★	★	-		★	-	5
Sasajima et al 2000	★	★	★	★	★		★	-	6
Van Keulen et al 2018	★	★	★	★	★		★	-	6
He et al 2019	★	★	★	★	★		★	-	6
Bowman et al 2019	★	★	★	★	★★		★	-	7
Jauk et al 2019	★	★	★	★	★★		★	★	8
Ocagli et al 2021	★	★	★	★	★		★	-	6
Fortini et al 2014	★	★	★	★	★		★		6
Laharya et al 2014	★	★	★	★	★		★		6
Bilge et al 2015	★	★	★	★	-		★		5
Chaiwat et al 2019	★	★	★	★	★		★		6
Xing et al 2019	★	★	★	★	★		★		6
Huang et al 2021	★	★	★	★	★		★	★	7
Li et al 2024	★	★	★	★	★★		★	-	7
Park et al 2017	★	★	★	★	★		★	-	6
Yangisawa et al 2022	★	★	★	★	★★		★	-	7
Sun et al 2023	★	★	★	★	★		★	-	6

Ishibashi-Kanno et al 2022	★	★	★	★	★★	★	-	7
Klimiec et al 2022	★	★	★	★	★★	★	-	7
Wang et al 2022	★	★	★	★	★★	★	-	7
Xiao et al 2022	★	★	★	★	★	★	-	6
~~Feng et al 2022~~	★	★	★	★	★	★	-	6
Yan et al 2024	★	★	★	★	★★	★	★	8
Rady et al 2005	★	★	★	★	★	★	-	6
Chang et al 2018	★	★	★	★	★	★	-	6
Vasa et al 2024	★	★	★	★	★★	★	-	7
Tian et al 2024	★	★	★	★	★★	★	-	7
Arrieta et al 2021	★	★	★	★	★★	★	-	7
Long et al 2024	★	★	★	★	★★	★	-	7
Zhang et al 2023	★	★	★	★	★★	★	★	8
Li et al 2024	★	★	★	★	★	★	★	7
Mondal et al 2022	★	★	★	★	★★	★	-	7
Liu et al 2022	★	★	★	★	★★	★	-	7
Gao et al 2022	★	★	★	★	★★	★	-	7
Paolisso et al 2022	★	★	★	★	★★	★	★	8
Wei et al 2022	★	★	★	★	★★	★	★	8
Shen et al 2021	★	★	★	★	★★	★	-	7
Guo et al 2021	★	★	★	★	★	★	-	6
Zhu et al 2019	★	★	★	★	★	★	-	6
Zhou et al 2017	★	★	★	★	★	★	-	6
Ozyurtkan et al 2010	★	★	★	★	★	★	-	6
Heymann et al 2007	★	★	★	★	★	★	-	6
Shirvani et al 2022	★	★	★	★	★	★	-	6
Windmann et al 2019	★	★	★	★	-	★	-	5
Lin et al 2021	★	★	★	★	★	★	-	6

Krzych et al 2014	★	★	★	★	★	★	-	6
Jang et al 2017	★	★	★	★	★★	★	-	7
Chu et al 2021	★	★	★	★	★	★	-	6
Liu et al 2021	★	★	★	★	★★	★	-	7
Mora-Garzon et al 2024	★	★	★	★	★	★	-	6
Wang et al 2024	★	★	★	★	★	★	★	7
Liu et al 2022	★	★	★	★	★	★	-	6
Zhou et al 2020	★	★	★	★	★	★	-	6
van Keulen et al 2018	★	★	★	★	★	★	-	6
Saager et al 2015	★	★	★	★	★	★	-	6
Zhao et al 2022	★	★	★	★	★	★	-	6
Gandhi et al 2005	★	★	★	★	-	★	-	5
Park et al 2024	★	★	★	★	★	★	-	6
Oh et al 2024	★	★	★	★	★	★	-	6
Song et al 2024	★	★	★	★	★	★	-	6

Supplementary Material Table S1

First Author and Year	Type of Article	Study Design	Aim of Study	Total Population	Diagnosis of Delirium	Impact of Diabetes or on delirium	Other Risk Factors	Main Results
Bucerius et al 2003	Original Research	Observational Prosective Study	Impact of diabetes on cardiac surgery	16184	American Psychiatric Associatio n Guidelines	p<0.0001	NA	Diabetes is a risk factor for stroke, renal insufficiency, sternal infection and/or instability, respiratory insufficiency, prolonged ICU stay.
Bucerius et al 2004	Original Research	Observational Prosective Study	risk factors for post-cardiac surgery delirium	16184	American Psychiatric Associatio n Guidelines	OR= 1.31 1.16-1.49 p<0.0001	Cerebrovascular disease, Peripheral vascular disease, LVEF<30%, Cardiogenic shock, Urgent operation, Operation time> 3h, Intraoperative hemofiltration, Transfusion > 2 L	Beating-heart surgery without cardiopulmonary bypass may lead to a lower prevalence of complications.
Bucerius et al 2005	Original Research	Observational Prosective Study	impact of diabetes on CABG surgery	9682	American Psychiatric Associatio n Guidelines	OR=1.33 95% CI 1.1-1.62 p=0.005	Renal Dysfunction,	Diabetes is an independent predictor of postoperative delirium, renal dysfunction and

								respiratory insufficiency
Yildizieli et al 2005	Original Research	Retrospective Study	incidence and risk factors for delirium	432	DSM IV	p=0.04	Age, Sleep deprivation Operation time, Markedly abnormal serum values	Older age, sleep deprivation, abnormal postoperative levels of sodium, potassium, or glucose, and longer operation time can prevent occur- rence of postoperative delirium
Kotfis et al 2018	Original Research	Retrospective Study	incidence and risk factors of delirium in elderly (≥65 years) and very elderly (≥80 years) patients undergoing major cardiac surgery.	1797	DSM 5	OR=1.346 95% CI 1.049-1.726 p=0.019	Age, EF<30%, Arteriopathy, AF Pneumonia, Postoperative creatine ,Hospital LOS	Independent risk factors for development of post-cardiac surgery delirium were age, low ejection fraction, diabetes, extracardiac arteriopathy, postoperative atrial fibrillation, pneumonia, elevated creatinine, and prolonged hospitalization time
Milisen et al 2020	Original Research	Secondary data analysis	Association between	190	CAM 20 items	OR=3.15 95% CI 1.42-7	Age, ADL Index, Time of	No relationship between

		of a randomized, observer-blind, controlled trial	preoperative anxiety and postoperative delirium in older patients undergoing cardiac surgery.				cardiopulmonary bypass,	preoperative anxiety and postoperative delirium.
Ordonez-Velasco and Hernandez-Leiva 2021	Original Research	Observational Study	perioperative risk factors independently associated with delirium	311	CAM-ICU	OR 3.5 95% CI 1.18-10.6	Very low/no education, Stroke/TIA, Type of surgery, Transfusion	age, comorbidities, and complexity of cardiac surgery are associated with delirium
Li et al 2021	Original Research	Retrospective Study	incidence and risk factors of delirium in elderly (aged ≥65 years) patients who underwent coronary artery bypass grafting (CABG).	1426	CAM-ICU	OR=1.51 95% CI 1.19-1.91 p=0.001	Age, Drinking, Stroke, ICA stenosis	Age, long-term alcohol consumption, diabetes, stroke, and carotid artery stenosis were independent risk factors for delirium in elderly patients who underwent CABG
Tan et al 2008	Original Research	Observational Study	ncidence and predictors of delirium after cardiac surgery.	53	American Psychiatric Association Guidelines	p<0.05	Left ventricular dysfunction, History of cerebrovasuclar disease	The strongest predictor of both incident delirium and delirium symptoms was a history of cerebrovascular disease

Nikolic 2012	Original Research	Observational Prosective Study	risk factors for postoperative delirium in cardiac coronary patietns	370	NA	$p<0.05$	Cerebrovascular disease, Peripheral vascular disease, and Prolonged intubation	diabetes mellitus, cerebrovascular disease, peripheral vascular disease, and prolonged intubation are risk factors for delirium
Smulter et al 2013	Original Research	Observational Study	risk factors for delirium in older patients undergoing cardiac surgery with cardiopulmonar y bypass	142	DSM-IV	OR=3.5 95% CI 1.1-11 p=0.032	Age, Gastric/ulcer problems, Volume load during operation, Ventilator time in ICU, Highest temperature recorded in ICU, Sodium concentration	delirium was strongly associated with an increased volume load during surgery.
Ding et al 2024	Original Research	Observational Study	identify the risk factors for postoperative delirium after cardiac surgery in frail elderly patients and development of a prediction model	130	NA	p=0.003	Frailty, MMSE, Operation time	frailty index, preoperative MMSE score, and operation time were independent risk factors for POD in elderly patients after cardiac surgery
Afonso et al 2010	Original Research	Observational Prosective Study	develop a predictive model for postoperative delirium using demographic	112	RASS; ICAM-ICU	NS	Age, Increased duration of surgery	Increased age and increased duration of surgery were independently associated with

			and procedural parameters.					postoperative delirium.
Wang et al 2015	Original Research	Observational Prosective Study	risk factors that contribute to the development of postoperative delirium in geriatric patients	200	NA	NS	Age, General anesthesia, Longer surgical duration (>3 hours), Intraoperative hypercapnia and Hypotension, Preoperative affective dysfunction, Postoperative sleep disorders	Advanced age , use of general anesthesia, longer surgical duration (>3 hours), the presence of intraoperative hypercapnia and hypotension, the presence of preoperative affective dysfunction, and the presence of postoperative sleep disorders are associated with postoperative delirium in geriatric patients after orthopedic surgery.
Mauri et al 2012	Original Research	Observational Prosective Study	incidence, risk factors, and association with long-term outcome of postoperative delirium after TAVR	661	RASS; ICAM-ICU	NS	Male sex, General anesthesia, AF, Vascular complication, Pneumonia, Frailty, Stroke	delirium is a frequent finding after TAVR and significantly associated with reduced 2-year survival

Miyazaki et al 2011	Original Research	Observational Study	advanced arteriosclerotic changes as a risk factor of TIA, delirium and stroke after off pump CABG	685	DSM IV	p<0.001 (only univariate analysis)	Age ≥75 years, Carotid artery stenosis, Creatinine, Hypertension, AF , Smoking	CAS >50%, was a significant predictor of postoperative stroke or TIA and delirium
Jodati et al 2013	Original Research	Observational Study	prevalence and risk factors for development of delirium in patients undergoing open heart surgery in , Iran.	328	NA	NS	Hypertension, AF, Lung disease	4.8% with risk factors such as older age, longer ICU stay, longer CPB time, and longer mechanical ventilation time developed delirium
Kotfis et al 2019	Original Research	Observational Prosective Study	occurrence of delirium in cardiac surgery is associated with diabetes or elevated preoperative HbA1c	3178	DMS 5	OR: 1.703, 95% CI: 1.401-2.071, p<0.001	Age, NYHA class III,IV, HbA1c% 1.2 Creatinine at admission 1.18 , Extracardiac arteriopathy	More diabetic patients develop delirium after cardiac surgery than nondiabetic patients. Elevated preoperative HbA1c level is a risk factor for postcardiac surgery delirium regardless of the diagnosis of diabetes.
He et al 2021	Original Research	Retrospective Study	To construct a prediction model for	438	DSM 5	OR=0.003 95%CI 0.000-0.019 p<0.001	Smoking, Previous	smoking, diabetes, previous cardiovascular

			delirium in patients with type A aortic dissection after surgery				cardiovascular surgery	surgery, EF, time to aortic block, acute kidney injury, low cardiac output syndrome, and pulmonary complications were included in a predictive model for delirium
Huang et al 2019	Original Research	Observational Study	incidence and risk factors for delirium following total joint arthroplasty	11970	ICD-9	OR=1.70, 95% CI 1.1.5–2.47, p = 0.009	Older age, Renal disease, Blood transfusions, and Sedation during anesthesia recovery	Dementia is strongly associated with POD. The association between POD and transfusions may reflect higher acuity patients or detrimental effect of blood.
Yang et al 2020	Original Research	Retrospective Study	incidence and risk factors associated with delirium after primary elective total hip arthroplasty	388424	ICD-9	uncomplicated diabetes OR=1.27 95% CI 1.15-1.39 p<0.001; complicated diabetes OR=1.27 95%CI 1.15-1.39 p<0.001	Age≥61, Female, Alcohol, Anemia Coagulopathy, Depression, Drug abuse, Hypertension, Lymphoma, Electrolyte disorder Obesity, Peripheral vascular disorders , Psychosis, Renal Failure, Valvular	Postoperative delirium of THA was associated with increased preoperative comorbidities, LOS, total charges, in-hospital mortality, and major perioperative complications including acute

							disease Weight loss	renal failure and pneumonia.
He et al 2020	Original Research	Observational Study	incidence and perioperative risk factors of delirium and investigate whether NLR could serve as a potential marker	680	DSM-IV; ICAM	OR=1.58 95% CI 1.06-2.36; p = 0.025	NLR ≥ 3.50, Age.	lder age, diabetes, higher neutrophil count, and NLR ≥ 3.5 were independent risk factors
Ma et al 2023	Original Research	Observational Prosective Study	to explore the association of preoperative frailty with delirium in elderly patients undergoing hip arthroplasty.	228	CAM	HR 2.889 95% CI 1.171-7.132 p=0.021	Age, Hypertension , Insomnia	Frailty is a crucial risk factor for delirium
Yang et al 2022	Original Research	Retrospective Study	incidence and risk factors of delirium after primary total knee arthroplasty	1228879	ICD-9	p<0.05	Advanced age, Neurological disorders, Alcohol and Drug abuse, Depression, psychoses, Fluid and electrolyte disorders, Weight loss, deficiency and Chronic blood loss Anemia, Coagulopathy, Congestive heart Hailure, Chronic pulmonary disease,	neurological disorders were found to have the strongest association with the occurrence of postoperative delirium.

							Pulmonary circulation disorders, Peripheral vascular disorders, Chronic renal failure, and Teaching hospital.	
Wang et al 2018	Original Research	Retrospective Study	incidence and related risk factors of delirium in elderly patients with hip fracture.	306	CAM	OR= 0.330 95% CI 0.132-0.822 p=0.017	Age, Albumin infusion, ASA	The elderly patients over the age of 75 years with the history of diabetes or ASA classification > 2 level were at higher risk of delirium
Ahn et al 2022	Original Research	Retrospective Study	incidence of delirium and the preoperative factors associated with perioperative use of drugs to treat hyperactive delirium in elderly patients who underwent hip fracture surgery under regional anesthesia.	58972	ICD-10	OR= 1.093 95% CI 1.041-1.148 p<0.0013	Age, Hospital type, Sex, Depression, Neurodegenerative disorders, Psychosis Peptic ulcers 0.916 Ventilator care	Hyperactive delirium requiring pharmacologic intervention patients who underwent surgery for hip fracture under regional anesthesia was associated with multiple risk factors, including male sex, old age, preoperative neurodegenerative disorder, diabetes mellitus, peptic

								ulcer disease, psychosis, depression, ICU stay, and ventilator care.
Haynes et al 2021	Original Research	Observational Study	Predictors of postoperative delirium and its association with preexisting dementia and adverse postoperative outcomes	18754	NA	OR=1.30 95%CI 1.06-1.59 p<0.001	Age ≥80, Female ASA≥3, Functional status, Bleeding disorder, Preoperative dementia.	Postoperative delirium is a potentially preventable postoperative adverse outcome that was seen in 18.8% of patients with hip fracture. Those with preoperative dementia seem to be a particularly at-risk subpopulation.
Wang et al 2021	Original Research	Retrospective Study	incidence and risk factors of postoperative delirium in elderly patients after hip fracture surgery	272	CAM	OR=7.51 95% CI 2.903-19.416 p<0.0001	Intraoperative lactic acid >2 mmol/L Bispectral index, ASA <II , VAS score>3, drinking history	risk stratification index (RSI) can safely guide postoperative outcomes of elderly patients with hip fractures, and RSI ≥ 5 may be able to predict the onset of postoperative delirium.

Venkatakrishnaiah et al 2022	Original Research	Observational Prosective Study	risk factors for the development of delirium in patients with hip fractures.	110	CAM	HbA1c > 7.5% OR=59.262 95% CI 4.972-706.370 p<0.001	Age, Malnutrition, Serum albumin <3.5g%	Advancing age, hypoalbuminemia, malnourishment, and uncontrolled diabetes are strong predictors for the development of delirium
Shang et al 2024	Original Research	Prospective matched cohort study	preoperative diabetes is associated with delirium after elective orthopedic surgery and intraoperative frontal alpha power is a mediator of the association between preoperative diabetes and delirium	266	CAM	OR= 3.2 95% CI 2 1.4-8.0; p = .009	Intraoperative alpha power	This study suggests that preoperative diabetes is associated with an increased risk of POD in older patients undergoing major orthopedic surgery, and that low intraoperative alpha power partially mediates such association
Zhang et al 2024	Original Research	Retrospective Study	Construction and validatation of a nomogram that predicts the likelihood of postoperative delirium	681	CAM	OR= 1.879 95% CI 1.121-3.149 p=0.017	Age, BMI, Education Barthel Index, Hemoglobin, CVD	The use of this novel nomogram can help clinicians predict the likelihood of delirium after hip arthroplasty in elderly patients
Shen et al 2022	Original Research	Retrospective Study	Construction and validate a	1312	CAM	diabetes with random	Preoperative delirium, CV	prediction score for postoperative

			prediction score of postoperative delirium in geriatric patients undergoing hip fracture surgery or hip arthroplasty.			glucose > 13 mmol/l OR=2.43 95% CI 1.32-2.99 ; glucose 8-13 mmol/l OR=1.36 95% CI 1.15-1.67 p=0.023	accident with modified Rankin scale, Age ≥80, Benzodiazepines , Surgical delay, Creatine ≥90 µmol/L Smoker status	delirium in geriatric patients undergoing hip fracture surgery or hip arthroplasty was derived and validated
Shih et al 2007	Original Research	Observational Study	risk factors for the development of confusion or delirium in patients with posterior cerebral arterial infarction	29	NA	p<0.05	Medial occipito-temporal gyri on the left side	involvement of the medial occipito-temporal gyri, especially on the left side was the pivotal factor for the development of confusion or delirium
Gao et al 2008	Original Research	Retrospective Study	risk factor for delirium in spinal surgery	549	DSM IV	OR=2.981 95% CI 0.912-9.746 p=0.07	CNS disorder, Surgical history, Age> 65	Older age, diabetes or central nervous system disorder, surgical history, blood transfusion ≥800 mL, and low concentrations of HCT/HGB on the first day after surgery are probable risk factors for postoperative DELIRIUM

								delirium in spinal surgery
Kolk et al 2022	Original Research	Observational Study	risk factors for delirium in head and neck surgery	100	NA	p<0.05	Age, TSH, free fibula transplant	free fibula transplant was a signficant predictor
Gold et al 2022	Original Research	Observational Study	risk factor for delirium in lumbar spinal fusion	702	CAM-ICU	p<0.05	Advanced age, Lower preoperative and postoperative hemoglobin, Higher ASA grade, Greater extent of surgery, and Higher postoperative pain scores.	Patients with delirium had a higher incidence of postoperative ICU admission, increased length of stay, decreased likelihood of discharge to home and increased mortality
Zheng et al 2024	Original Research	Observational Study	risk factors for delirium in lumbar spinal fusion	493481	NA	p<0.05	Advanced age (≥ 65 years), Pre-existing neurological disorders, alcohol or drug abuse, Depression, Psychotic disorders, Fluid and electrolyte imbalances, Weight loss, Anemia, Coagulopathy, Congestive heart failure, Pulmonary	neurological disorders demonstrated the strongest correlation with delirium

							Peripheral vascular disease, Chronic renal insufficiency, and Receiving treatment at a teaching hospital	
Visser et al 2015	Original Research	Observational Prosective Study	Incidence of and risk factors for delirium in electively treated vascular surgery patients	566	DSM	$p<0.05$	Cognitive impairment open aortic surgery or amputation surgery Current smoking, Hypertension 7.6; and Age ≥80 years	cognitive impairment and open aortic or amputation surgery were highly significant risk factors for delirium, which was sassociated with a higher mortality and more institutionalization .
Sasajima et al 2000	Original Research	Observational Study	incidence and specific markers of postoperative delirium in elderly patients with chronic lower limb ischaemia	110	CAM	$p=0.234$	Age≥ 70, Critical limb ischemia.	The incidence of postoperative delirium in elderly patients with chronic lower-limb ischaemia was high and age of over 70 years and critical limb ischaemia were identified as specific markers

van Ejseden et al 2015	Original Research	Observational Study	risk factors for delirium in patients with critical limb ischemia undergoing surgery and outcome	92	DOSS	OR=6.23 95% CI 1.11-52.2; p=0.035	Short Nutritional Assessment Questionnaire for Residential Care ≥3	Delirium is a common adverse event in patients with critical limb ischemia undergoing surgery with devastating outcome in the long term.
van Keulen et al 2008	Original Research	Observational Study	association between diabetes and glucose dysregulation with ICU delirium.	2745	DSM	OR=0.93 95% CI, 0.73-1.18	NA	Hyperglycemia and the occurrence of hyperglycemia and hypoglycemia on the same day were associated with ICU delirium but only in patients without diabetes.
He et al 2019	Original Research	retrospective Study	risk factors for postoperative delirium (PODE) in patients undergoing microvascular decompression	912	DSM-5	p=0.029	Male sex, Hypertension, Sleep distrubance, Mount Fuji sign	Old age, male sex, hypertension, preoperative carbamazepine use, postoperative sleep disturbance, and tension pneumocephalus are associated with delirium
Bowman et al 2020	original Research	Retrospective case-control study	risk factors for delirium occuring at community or	85607	Inpatient Records	NS	Cognitive impairment or mental illness, Prior delirium,	risk factors in primary care overlap with predictors of

			emergency admission				Psychoactive drugs, frailty/related conditions, Infection markers, Metabolic disturbance, Hyponatraemia and High anticholinergic burden	delirium in hospital inpatients.
Jauk et al 2018	Original Research	Retrospective Study	influence of incorrect administrative diabetes coding on the performance of a risk prediction model for deliriu		Records coding	NA	NA	no significant impact of incorrect diabetes coding on the performance for our model predicting delirium.
Ociagli et al 2018	Original Research	Observational Study	predictors that are mostly associated with the risk of delirium episodes using a machine learning technique	78	4AT scale	p=0.52	Dementia, Hearing impairment.	The use machine learning of this model may allow for early detection of delirium onset
Fortini et al 2014	Original Research	Observational Prosective Study	impact of delirium on geriatric inpatients in internal medical	560	CAM	OR 1.936 p<0.005	Cognitive impairment on admission, Chronic kidney	elirium impact is relevant to older patients hospitalized in internal medicine

			wards and to identify predisposing factors				failure, Male gender	wards and cognitive impairment is a risk factor for incident delirium.
Lahariya et al 2014	Original Research	Observational Study	incidence, prevalence, risk factors and outcome of delirium in patients admitted to a cardiac intensive care unit	309	CAM	diabetes , uncontrolled diabetes	Hypokalemia, Hyponatremia, Sequential Organ Failure Assessment score, Cognitive deficits, Receiving more than three medications, Sepsis, Cardiogenic shock, CABG, EF <30%, Opioids, Age > 65, Seizures, AF, Depression, Benzodiazepines, Warfarin, Ranitidine, Steroids, Higher total number of medications, Anaemia, Hypoglycemia, Charlson Comorbidity Index score.	Delirium is prevalent in cardiac insensive care unit setting and incrises mortality and longer hospital stay.

Bilge et al 2015	Original Research	Observational Study	risk factors and the incidence of delirium in patients who were followed postoperatively in our surgical intensive care unit for 24 h	250	CAM	OR= 2.736 95% CI 1.360-5.501 p=0.005	Age, ASA score, COPD, Duration of operation	lder age, high ASA score, preoperative DM and COPD are important risk factors for the development of delirium. Regional anaesthesia, high postoperative pain scores and meperidine use were observed to be associated with the development of delirium
Chaiwat et al 2019	Original Research	Observational Study	incidence of, and the risk factors for delirium in surgical intensive care unit	250	CAM-ICU	OR= 3.03 95% CI 1.43–6.44 p=0.004	Age, SOFA, Mechanical ventilation, Benzodiazepine use	A risk score utilizing 6 variables was able to predict which patients would develop delirium
Xing et al 2019	Original Research	Observational Study	develop and validate a postoperative delirium prediction model for ICU	400	CAM-ICU	3.415 95% CI 1.531-7.614 p= 0.002	Coma, Hypertension, Acid-base imbalance, POSSUM score	The model, which used readily available data, exhibited high predictive value AUC:0.852
Huang et al 2021	Original Research	Observational Study	develop a predictive model for delirium after	800	CAM-ICU	OR=2.541 95% CI 1.201-5.377 p=0.015	Age, Education level <9 years, Smoking, Supratentorial lesions,	The E-PREPOD-NS model can predict delirium AUC :0.851

			elective craniotomy				Anesthesia duration > 6 h, GCS < 9pts, Metabolic acidosis	
Li and Guo 2024	Original Research	Retrospective Study	risk factors for delirium in ICU and application of emotional nursing with pain nursing in the management of delirium	301	CAM-ICU	OR=4.631 95% CI 1.787-11.999 p=0.002	Emotional and pain care, Emergency, smoking APACHE score, ICU stay	Patients with diabetes and or smoking history, postoperative patients, patients with a high APACHE II score, and those with emergency ICU admission need emotional and pain care.
Park et al 2017	Original Research	Observational Study	incidence and risk factors for delirium in patients that underwent liver resections.	196	DSM-IV	p<0.05	Age, Serum albumin level, Presence of cerebrovascular disorder, Use of benzodiazepines, and a Previous history of delirium.	age, serum albumin level, presence of cerebrovascular disorder, use of benzodiazepines, and a previous history of delirium are risk factors for delirium
Yanagisawa et al 2022	Original Research	Retrospective Study	relationship between preoperative physical activity and posto operative delirium in	178	CAM	OR= 0.47 95% CI 0.15−1.48 p=0.2	Gait speed, Physical activity	Low physical activity is a predictor of delirium in patietns with gastrointestinial cancer

			gastrointestinal cancer patients.					
Sun et al 2023	Original Research	Observational Study	relationsheep between CRP and post-operative delirium in elderly withcolorectal cancer	643	CAM	OR=2.138 95% CI 1.214–3.766 p=0.008	Age, COPD, CRP	postoperative CRP is associated with delirium onset
Ishibashi-Kano et al 2020	Original Research	Retrospective Study	risk factors for postoperative delirium after oral tumor resection and reconstructive surgery.	69	CAM	HR 4.872 95% CI 1.153–20.576	Sedation period	Sedation period was factor related to post- operative delirium.
Klimiec et al 2017	Original Research	Observational Study	association between pre-stroke neuropsychiatric symptoms and the risk of delirium in stroke patients	606	DSM-5	p<0.01	Neuropsychiatric Inventory subscale, apathy	Pre-stroke apathy symptoms are associated with increased risk of delirium in stroke patients.
Wang et al 2022	Original Research	Observational Study	relationship between cardiovascular disease risk score and postoperative delirium among patients with	750	CAM	NA	FHS–CVD risk score, T-tau, and P-tau	Higher vascular risk score is one of the preoperative risk factors for delirium partly mediated by CSF biomarker tau protein.

			total knee arthroplasty					
Xiao et al 2023	Original Research	Retrospective Study	To determine the incidence of delirium and its related risk factors in patients with senile dementia during hospitalization	157	CAM	OR= 4.99 95% CI 1.93-12.93 p<0.01	CV disease, VAS score ≥4 pts, sedative drugs, Sodium <129 U/mL	Diabetes, cerebrovascular disease, VAS ≥4 points, use of sedative drugs, and Sodium levle <129 U/mL were independent risk factors for delirium in patients with dementia.

Supplementary Material Table S2. Delirium and Diabetes
OR: Odds Ratio, CI: Confidence Interval

First Author and Year	Type of Article	Study Design	Aim of Study	Total Population	Defintion of Stress hyperglycemia	Impact of Stress Hyperglicemia on Mortality	Other Risk Factors for poor glycemic control	Impact of Stress Hyperglycemia on Hospitalization length	Other Results
Dunkan et al 2009	Review	Narrative Review	Classification of stress hyperglycaemia, mechanisms of harm, and management strategies.	NA	Fasting glucose >6·9 mmol/L or random glucose >11·1 mmol/L without evidence of previous diabetes), and pre- existing diabetes with deterioration of preillness glycaemic control.	2.7-18.3 higher risk	NA	NA	Stress hyperglycemia has poor outcome in ICU, CVD and stroke, surgery setting.
Yan rt al 2024	Original Research	Retrospective Study	Association between the SHR and adverse outcomes in critically ill patients with sepsis	2312	SHR	In-hospital mortality quartile 4: HR= 1.84 95% CI 1.32–2.58 P<0.001 : 28 days HR quartile 4: 95% CI 1.84 1.38–2.4 p<0.001	NA	NA	The SHR can be used to predict adverse outcomes in critically ill patients with sepsis

Li et al 2024	Review	Meta-analysis	To explore the association between SHR and the incidence of adverse clinical events with heart failure (HF)	15250	SHR	RR=1.61, 95%CI 1.17-2.21, p = 0.003	NA	NA	Increased rehospitalization RR: 1.83, 95% CI: 1.44-2.33, p < 0.001; Higher incidence of cardiovascular death RR: 2.19, 95% CI: 1.55-3.09, p < 0.001;Major adverse cardiovascular events RR: 1.54, 95% CI: 1.15-2.06, p = 0.004;
Capes et al 20201	Review	Systematic Overview and Meta-analysis	Stress hyperglycemia and prognosis of stroke in nondiabetic and diabetic patients	32 studies	Individual studies	RR= 3.07 95% CI 2.50- 3.79 in nondiabetic patients RR= 1.30 95% CI, 0.49-3.43 in diabetic patients (ischemic or hemorragic stroke)	NA	NA	Nondiabetic stroke survivors whose admission glucose level was >6.7 to 8 mmol/L also had poor functional recovery RR:1.41 95% CI, 1.16 - 1.73
Abdelhamid et al 2016	review	Systematic Review and Meta-analysis	the role of stress hyperglicemia on diabetes	2923	Individual studies	NA	NA	NA	Increased risk of incident diabetes OR 3.48 95 % CI 2.02-5.98
Rady et al 2005	Original Research	Case-control study	Relationship of patient and critical illness characteristics	7285	Fasting glucose level	NA	advanced age, history of diabetes,	NA	Compared with nondiabetic survivors, nondiabetic

			to glycemic control with insulin and hospital survival.				cardiac surgery, postoperative complications, severity of illness, nosocomial infections, prolonged mechanical ventilation, or concurrent medications.		nonsurvivors had longer periods with glucose levels greater than 144 mg/dL. Diabetic nonsurvivors vs diabetic survivors had longer periods with glucose levels greater than 200 mg/dL.
Chang et al 2018	Original Research	Cross-sectional study	Role of stress hyperglycemia on outcome in trauma patients	2482	Serum glucose level of ≥200 mg/dL	Increased: OR=5.12 95% CI 3.72–7.05; p < 0.001			Longer ICU stay 10.1 vs. 6.7 days (SH vs diabetic normalglycemia) p < 0.001
Vasa et al 2024	original Research	Retrospective Study	SH and length of hospitalization after endoscopic intracerebral hemorrhage evacuation	192	Preoperative glucose-to-HbA1c ratio > calculated-median	NA	NA	Longer ICU stay :OR=2.44; p=0.026	SH compared to non SH was more likely to have a history of diabetes (43 % vs. 27 %, p=0.034), Intraventricular hemorrhage (54 % vs. 33 %, p=0.007), higher

									preoperative hematoma volumes (46.8 ml vs. 38.6 mL, p=0.02), higher postoperative hematoma volumes (6 ml vs. 2.9 mL, p=0.008), smaller evacuation percentages (86.7 % vs. 92.7 %, p=0.048), longer procedure lengths (2.78 hrs vs. 2.23 hrs, p=0.015), and prolonged ICU LOS (9.44 days vs. 5.68 days, p=0.003)
Tian et al 2024	Original Research	Retrospective Study	The prognosis of SH on critically ill patients with cardiogenic shock.	393	SHR	HR= 1.511 95% CI 1.124- 2.030 p=0.006	Age, HF	NA	SHR had no significant effect on the prognosis of CS patients after 28 days, 90 days, or 1 year.
Arrieta et al 2021	Original Research	Observationial Study	The role tress hyperglycemia and osteocalcin on the prognosis of COVID-19 critically ill patients	52	plasma glucose level of ≥140 mg/dl.	p=0.085	NA	NA	SH in COVID-19 patients was associated with higher amounts of glucose delivered through artificial nutrition

Long et al 2024	Original Research	Observational Study	The association of stress hyperglycemia with 90-day all-cause mortality in intensive care unit in pulmonary hypertension	414	ratio of admission glucose to HbA1c (GAR)	GAR Quartile 4 HR= 2.73 95% CI 1.21-6.17	elderly patients (≥ 70 years: HR 4.22, 95% CI: 1.98-8.99), female patients (HR 4.36, 95% CI: 1.62-11.72), and patients with an HbA1c level ≥ 6% (HR 5.28, 95% CI: 2.07-13.46)	Increased (p=0.003)	Significant correlation between the severity of disease and the increase in blood glucose when patients with pulmonary experience stress.
Zhang et al 2023	Original Research	Retrospective Study	The relationship between SHR and the short- and long-term prognoses of ICU	3887	SHR	in hospital mortality: SHR OR=2.92 95% CI 2.14-3.97]P < 0.001; 1 year follow-up: HR= 1.55 95% CI 1.26-1.90 p < 0.001	SIRS Score, SOFA score, Meld score, APSIII Score, LODS Score, Oasis Score.	NA	SHR had an incremental effect on various illness scores. Non-diabetic patients, rather than diabetic patients, showed an increased risk of all-cause mortality.

Li et al 2024	Original Research	Retrospective Study	SHR and all-cause mortality in cardiac ICU	5564	SHR	SHR> 0.95 OR= 1.41 95% 1.25-1.59; SHR<0.95 OR= 0.56, 95% CI: 0.34 to 0.91	NA	NA	The inflection point of SHR for poor prognosis was identified at an SHR value of 0.95.
Mondal et al 2022	Original Research	Observational Study	The role of Admission blood glucose and SH on mortality in COVID-19 patients with type 2 diabetes	451	SHR, ABG	SHR ≥ 1.14 OR: 7.81,4.07-14.98	NA	SHR ≥ 1.14 OR: 4.41 2.49-7.84	AUROC of SHR in predicting mortality was significantly higher than ABG in all subgroups
Liu et al 2023	Original Research	Observational Study	the association of SHR with all-cause mortality in critical AMI patients	4337	SHR	American cohort: SHR of quartile 4 HR = 1.87; 95% CI 1.40-2.50 (1 year mortality; HR = 1.63; 95% CI: 1.27-2.09 long term mortality; Chinese cohort HR: 1.44; 95%CI: 1.03-2.02 (1-year mortality) ; HR = 1.32; 95%CI: 1.05-1.66 (long term mortality).	NA	NA	SHR was significantly associated with higher 1-year and long-term all-cause mortality among patients without diabetes in American and Chinese population
Gao et al 2023	Original Research	Observational Prospective Study	Prognostic role of SHR on MI without	1179	SHR	SHR 1 SD HR= 2.33 95%CI 1.07–5.08 p=0.033 (death, non fatal	NA	NA	SHR is a better predictor of prognosis than admission

			obstructive CAD			stroke, revascularization) : SHR HR 1 SD HR= 2.30 95%CI 1.21–4.38 p=0.011 (MACE)			glycemia alone, especially in diabetic patients
Karakasis et al 2024	Review	Systematic Review and Meta-analysis	The prognostic value of SHR in patients with AMI	87974	SHR	In-hospital all-cause mortality OR = 3.87; 95 % CI 2.98-5.03 p < 0.001.	NA	NA	Higher SHR levels were predictors for MACCE :HR = 1.7; 95 % CI= [1.42, 2.03]; P < 0.001 in both obstructive and non obstructive CAD
Paolisso P. et al 2022	Original Research	Observational Study	investigated the link between stress hyperglycemia, inflammatory burden, and infarct size in a cohort of type 2 diabetic patients presenting with AMI treated with SGLT2-I versus other oral anti-diabetic (OAD) agents.	583	Admission Glycemia	NA	NA	NA	SH was lower among SGLT2I users compared to non-users(p=0.006)
Wei et al 2023	Original	Observational Study	various markers of stress	1099	Admission blood glucose	in-hospital death (ABG OR: 1.27	NA	NA	the risk significantly

	Research		hyperglycemia, such as admission blood glucose (ABG), fasting blood sugar (FBS), and stress hyperglycemia ratio (SHR) with different definitions, and the occurrence of adverse cardiovascular events in patients diagnosed with ST-elevation myocardial infarction (STEMI)		(ABG), fasting blood sugar (FBS), and stress hyperglycemia ratio (SHR) SHR1: ABG/ by the estimated average glucose level. SHR2, FBS/ by the HbA1c level. SHR3, FBS /the estimated average glucose level.	95% CI 1.19-1.36; FBS OR: 1.25 95% CI 1.16-1.35; SHR1 OR: 1.61 95% CI 1.21-2.14; SHR2 OR: 1.57, 95%CI 1.22-2.01; SHR3 OR: 1.59, 95%CI 1.24-2.05) and all-cause mortality (ABG HR: 1.10, 95% CI 1.07-1.14; FBS HR: 1.12, 95 CI 1.07-1.17; SHR1 HR: 1.19 95% CI 1.03-1.39; SHR2 HR: 1.28, 95%CI 1.14-1.44; SHR3 HR: 1.29, 95%CI 1.14-1.45			increased when the ABG and FBS levels exceeded 5mmol/L. Moreover, the inflection point for SHR was estimated to be 1.2.
Zhang et al 2023	Review	Meta-analysis	The incidence of SH in Acute Ischemic Stroke	4552	Individual studies	NA	NA	NA	SIH was 9.76–48.22%, and the pooled incidence was 24% 95% CI: 21–27%.
Shen et al 2022	Original Research	Observational Prospective Study	Stress hyperglycemia and clinical outcomes in patients with acute ischemic stroke treated	341	SHR1, fasting glucose (mmol/L)/glycated hemoglobin (HbA1c) (%); SHR2, fasting	NA	NA	NA	SHR1 [odds ratio (OR) 14.639, 95% CI, 4.075-52.589; P = 0.000] and SHR2 (OR, 19.700; 95% CI; 4.475-86.722;

			with intravenous thrombolysis		glucose (mmol/L)/[(1.59 × HbA1c)-2.59]; SHR3, admission blood glucose (mmol/L)/[(1.59 × HbA1c)-2.59]				P = 0.000) were independently associated with an increased risk of poor functional outcome
Guo et al 2021	Original Research	Observational Study	risk of stroke recurrence among non-diabetes mellitus (non-DM), previously diagnosed diabetes mellitus (PDDM), newly diagnosed diabetes mellitus-related hyperglycemia (NDDM-RH) and stress hyperglycemia after minor stroke or TIA	3026	SH:1) fasting plasma glucose≥ 7.0 mmol/L, using medications to decrease blood sugar levels for any reason during hospitalization,;2) Glycated albumin <15.5 %	NA	NA	NA	SH had a 9.7-fold risk of 90-day stroke recurrence HR =9.66, 95% CI 6.39-14.59, p < 0.001
Zhu et al 2019	Original Research	Observational Study	relationship between stress hyperglycemia and outcome of non-diabetic	999	SHR: FPG/ HbA1c.	SHR highest quartile vs lowest quartile: HR=2.86, 95%CI: 1.38-5.90 (12	NA	NA	SHR highest quartile vs lowest quartile HR = 2.19, 95% CI

			patients with acute ischemic stroke			months follow-up)			1.26-3.83 (stroke recurrence).
Zhou et al 2017	Original Research	Observational Study	stress hyperglycemia on the early vascular cognitive impairment (VCI) in stroke patients	422	Mild stress hyperglycemia group :6.1-7.0 mmol/L ,and the severe stress hyperglycemia group ≥7.0 mmol/L	NA	NA	NA	SH was an independent risk factor for VCI in patients with non-diabetic ischemic stroke OR=3.086,95% CI=1.065-8.929

Supplementary Material Table S3. Stress Hyperglycemia and Outcome
OR: Odds Ratio, CI: Confidence Interval; HR: Hazard Ratio

First Author and Year	Type of Article	Study Design	Aim of the study	Diagnosis of Delirium	Diagnosis of SH/hyperglycemia	Total Population	Delirium Incidence	SH and Delirium	Other Results
Heymann et al 2007	Original Research	Observational Prospective Study	To determine an association between hyperactive delirium and blood glucose levels in ICU patients.	Delirium Detection Score	Glycemia level in blood gas analysis every 4 h	196	28%	Adjusted OR= 4.152 95% CI 1.430-12.054 p=0.009	Delirium patients presented higher overall complication rates, length of ventilation, ICU stay and mortality rates .
Shirvani et al 2022	Original	Observational Study	To determine the clinical outcomes of	Neecham	Intraoperative glycemia level	90	47.7%	Adjusted OR= 1.01 95% CI	Aging, preoperative narcotic consumption,

	Research		CABG patients with delirium	confusion scale				1.006-1.028 p=0.002	diabetes and prolonged intubation are risk factors of delirium after CABG.
Windmann et al 2019	Original Research	Observational Study	To investigate the influence of intraoperative hyperglycemia on incidences of postoperative delirium postoperative cognitive dysfunction.	DSM-5	Blood glucose levels ≥150 mg/dL	87	47.1%	Adjusted OR 3.86 95% CI 1.13-39.49 p=0.044	SH was not associated with postoperative cognitive dysfunction (OR=3.59 p=0.157). Relative duration of hyperglycemia was higher in non diabetic patients with delirium (p=0.003).
Lin et al 2021	Original Research	Observational Study	to investigate the association of blood glucose variability with postoperative delirium in acute aortic dissection patients	CAM-ICU	SD of glucose level monitoring during 48 h	257	40.8%	Adjusted HR=1.418 95%CI 1.195-1.681 p=0.001	AUC for SD glood glucose 0.763 95% CI 0.704-0-821 p<0.001
Krzych et al 2014	Original Research	Cross-validation study	To validate a predictive scoring system for postoperative delirium in	DSM-4	Fasting glucose > 144 mg/dL	5781	4.1%	Fasting glucose > 144 mg/dL significant role	Both pre- and perioperative tools (DESCARD) had an excellent overall diagnostic accuracy (area under receiver operator

			cardiac surgery patients						characteristics curve = 0.83 and 0.89, respectively
Jang et al 2017	Original Research	Observational Study	Major delirium-causing risk factors in rehabilitation patients in subacute and acute stages of various neurological or musculoskeletal disorders	DSM-5	Blood glucose	398	16.3%	Higher glycemia level in deleirum vs non delirium patietn (p<0.001)	Depression, musculoskeletal disorders, traumatic brain injury, elevated WBC, BUN, AST, and CRP levels, and decreased potassium and phosphorus levels were identified as independent risk factors for delirium.
Chu et al 2021	Original Research	Observational Study	To evaluate the associated risk factors of postoperative delirium in patients with hip fracture	CAM	Blood glucose > 8.05 mmol/L	462	16.2%	Blood glucose > 8.05 mmol/L predicts delirium AUC= 0.792 0.723-0.829	Patients with a history of delirium, postoperative hypoxemia, blood glucose ≥8.05 mmol/L, albumin ≤32.26 g/L, and BMI ≤19.35 kg/m2 particularly need the attention of healthcare providers for the prevention of delirium.
Liu et al 2022	Original Research	Observational Prospective Study	To investigate whether the occurrence of postoperative delirium in	CAM	SH (1) no previous history of diabetes, (2) admission FBG ≥ 7 mmol/L, and (3)	309	16.83%	SH: p=0.834; preoperative FBG adjusted	The numbers of pre-injury physical performance and type 2 diabetes mellitus (T2DM)

			older hip fracture surgery patients is associated with preoperative glycemic control factors or pre-injury physical performance.		normal HbA1c values			OR= 0.804 95% CI 0.692-0.935 p=0.004.	patients were significantly different in the POD and non-POD groups.
Mora Garzon et al 2024	Original Research	Cross-Sectional Study	To assess the prevalence of poor glycemic control and its association with in-hospital adverse outcomes.	NA	Average of glucose capillary measurment < 100; > 180 mg/dl; Coefficient of variation >36%; Time in Range <70%	330	32.1%	OR 2.40 (1.29–4.43) p 0.005	Poor glycemic control, has been found to be associated with increased mortality and length of hospital stay.
Wang et al 2024	Original Research	Observational Study	To explore the associations of different blood glucose-related indexes and blood glucose change trajectory with postoperative delirium	CAM-ICU	Mean blood glucose (MBG), mean absolute glucose (MAG), mean amplitude of glycemic excursions (MAGE), glycemic lability index (GLI), largest amplitude of glycemic excursions (LAGE), and blood glucose trajectory in 24 h	1951	9.2%	MBG severe hyperglycemia adjusted OR=3.703 95%CI 1.743-7.870 p<0.001; MAG OR =1.754 95%CI 1.235–2.490 p=0.002; GLI OR= 1.458	The positive associations of MBG, MAG, and GLI with delirium were observed in patients aged <65 years old, male patients, White patients, those with eGFR <60 and INR <1.5, patients with sepsis, and those who received mechanical

								95%CI 1.033–2.058 p=0.032	ventilation and vasopressors (all p < 0.05).
Liu et al 2022	Original Research	Observational Study	To explore the relationship between preoperative fasting blood glucose levels and post operative delirium in non-diabetic older patients undergoing total hip replacement.	CAM, MDAS	Fasting plasma glucose	588	10.2%	Adjusted OR=1.427 95%CI 1.117–1.824 p=0.004	Preoperative FBG levels were negatively correlated with the CSF Aβ42 level (β = −0.290, P = 0.028)
Zhou et al 2020	Original Research	Observational Study	To evaluate the effects of isolated impaired fasting glucose (IIFG) on brain injury in patients undergoing cardiopulmonary bypass surgery.	NA	Impaired FG 5.6–6.9mmol/l, the 2-h post- prandial blood glucose level in the oral glucose tolerance test was <7.8mmol/l, and normal fasting blood glucose Level was <5.6mmol/l.	50	6%	NA	The increased postoperative NSE and S100B levels in the impaired fasting glucose compared with controls may be associated with severe insulin resistance and stress hyperglycemia.
van Keulen et al 2018	Original Research	Observational Study	To determine whether glucose variability is altered during	CAM-ICU	Glucose varability; mean glucose levels, MAG: mean aboulute glucose change/h;hyperglyce	2669	15.5%	Hypoglycemia in non diabetic adjusted OR=2.78	Delirium in critically ill patients with diabetes was associated with hypoglycemia

			delirium days compared to non-delirious days in critically ill patients with and without diabetes		mia > 8.0 mmol/l, and severe hyperglycemia > 11.0 mmol/l			95%CI 1.71-6.32 p=0.005	
Saager et al 2015	Original Research	Randomized Controlled Trial	To determine the effect of tight intraoperative glucose control using a hyperinsulinemic-normoglycemic clamp approach on postoperative delirium in patients undergoing cardiac surgery.	CAM	Blood glucose concentration>150 mg on day of surgery and>120 mg on susbequent postoperative days	198	20.7%	Tight glucose control RR 1.89, 95%CI 1.06–3.37; p = 0.03	Delirium severity, among patients with delirium, was comparable with each glucose management strategy.
Gandhi et al 2005	Original Research	Retrospective Study	To estimate the magnitude of association between intraoperative hyperglycemia and perioperative outcomes in	Definition based on Society of Thoracic Surgero	Intraoperative glucose level:mean, initial, maximal	409	5.4%	NA	For each 20-mg/dL increase in glucose concentration above 100 mg/dL a 34% increase in the likelihood of experiencing an event including: death, infection,

			patients who underwent cardiac surgery.	n criteria					pulmonary renal, stroke and delirium
Park et al 2004	Original Research	Retrospective Study	High preoperative blood glucose level and HbA1c level to determine the prevalence of postoperative delirium.	CAM	Fasting blood glucose level above 140 mg/dl (7.77 mmol/L) or a random blood glucose level above 180 mg/dl (9.99 mmol/L) within 24 h before surgical incision	23532	4.1%	Acute hyperglycemia: adjusted HR 1.33 95%CI 1.10-1-62 p=0.004; chronic hyperglycemia adjusted HR 0.94 95%CI 0.74-1.19 p=0.06	Acute and chronic hyperglycemia were associated with increased risk for 1 year and 3 years mortality.
Oh et al 2024	Original Research	Observational Study	To investigate the association between glucose dysregulation and delirium after non-cardiac surgery	CAM	Hyperglycemia was defined as at least one fasting blood glucose level >140 mg/dL or random glucose >180 mg/dL within 24 h before surgical incision	61805	3.1%	Hyperglycemia adjusted HR 1.36 95% CI,1.06-1.75	Exposure to hypoglycemia or both to hypo- and hyperglycemia was not associated with delirium in diabetic patients
Song et al 2022	Original Research	Observational Prospective Study	To determine the association between stress hyperglycemia and delirium.	CAM	SHR FBG / estimated average glucose	487	10.3%	SHR first tertile HR=3.71, 95% CI] 1.45-9.51; SHR third tertile HR =2.97, 95% CI 1.29-6.81	Relationship between SHR and delirium was more apparent in patients with HbA1c <6.5%, with significantly higher HR in the first and third SHR

									tertiles compared to the second tertile.
van Keulen et al 2018	Original Research	Observational Prospective Study	To investigate whether diabetes and glucose dysregulation (hyperglycemia and/or hypoglycemia) are associated with ICU delirium.	CAM-ICU	Blood glucose> 8mmol/l	2745	58.9%	Hyperglycemia OR=1.35; 95% CI, 1.15-1.59	Hyperglycemia and the occurrence of hyperglycemia and hypoglycemia on the same day were associated with ICU delirium but only in patients without diabetes.

Supplementary Table S4. Stress Hyperglycemia and Delirium
OR: Odds Ratio, CI: Confidence Interval
